# Earliest evidence of marine habitat use by mammals

**DOI:** 10.1038/s41598-021-88412-3

**Published:** 2021-05-13

**Authors:** Anton F.-J. Wroblewski, Bonnie E. Gulas-Wroblewski

**Affiliations:** 1 Department of Geology and Geophysics, University of Utah, 15 S 1460 E, Salt Lake City, UT 84112 USA; 2 Texas A&M Natural Resources Institute, 578 John Kimbrough Blvd. #2260, College Station, TX 77843 USA

**Keywords:** Ecology, Evolution, Zoology, Ecology

## Abstract

Evidence for the earliest invasion of the marine realm by mammals was previously restricted to Eocene (48.6–37.8 Ma) skeletal remains. We report incontrovertible ichnofossil evidence for brackish-water habitat use by at least two mammalian species in southern Wyoming during the late Paleocene (58 Ma). These are the first Paleocene mammal trackways recorded in the United States and only the fourth documented in the world. Multiple tracks preserved in restricted marine deposits represent animals repeatedly walking across submerged to partially emergent tidal flats. Hundreds of tracks are preserved in planform and cross-sectional exposure within five horizons along a 1032 m tracksite. Four prints exhibit five clear toe imprints, while two others distinctly display four toes. Some tracks penetrate beds populated by dwelling traces of marine bivalves and polychaetes in the upper layers and sea anemones at the base. Candidates for the five-toed tracemakers are pantodonts such as *Titanoides*, *Barylambda*, and *Coryphodon*, which have been recovered from late Paleocene strata throughout western North America. The four-toed tracks provide the earliest evidence of previously-undescribed large artiodactyls and/or tapiroids, mutually supporting recent molecular phylogenetic studies that place the origin of Cetartiodactyla near the Cretaceous-Paleogene boundary (~ 67.7 Ma). Collectively, these trackways irrefutably demonstrate the utility of ichnological data in reconstructing the evolutionary history and adaptive behaviors of extinct taxa beyond the evidence provided by body fossils alone.

## Introduction

Despite intense interest in and field studies of early Cenozoic mammalian radiation and dispersal patterns, Paleocene mammal trackways are exceedingly rare with only three previously reported worldwide. The early Paleocene *Sarjeantipes*^1^ from Alberta, Canada is a medium-sized, five-toed ichnotaxon that superficially resembles prints made by modern raccoons (*Procyon lotor*). A heavily-eroded 11.2 m long trackway, also from Alberta, Canada, was laid down by large mammals in the late Paleocene. Although the prints are reported to be wide-gauge^2^, they might represent a parallel pair of narrow-gauge tracks. A narrow-gauged trackway from the late Paleocene of Spitsbergen, Norway is the ichnotype of *Thulitheripus svalbardii* and has been attributed to large pantodonts traversing a continental coastal mire^3^.

Here we report a newly discovered, aerially-extensive series of late Tiffanian (58 Ma) mammalian trackways, dispersed across multiple stratigraphic intervals and traceable for 1032 m along a belt of well-exposed siltstone and very fine-grained sandstone outcrops. Uniquely preserved in brackish-water delta complexes within a restricted marine embayment or lagoon, these trackways attest to the recurrent use of marine habitats by medium- to large-bodied mammals during the late Paleocene. A minimum of two mammalian taxa are identifiable: one associated with relatively large, narrow-gauge, five-toed tracks, and the other with medium-sized, four-toed tracks. This direct evidence of marine habitat utilization by early Paleocene mammals predates the earliest mammalian skeletal remains preserved in marine sediments^4^ by 9.4–20 million years and highlights the potential for ichnological data to identify previously unknown taxa and their ecological adaptations.

## Geological context

**Figure 1:**
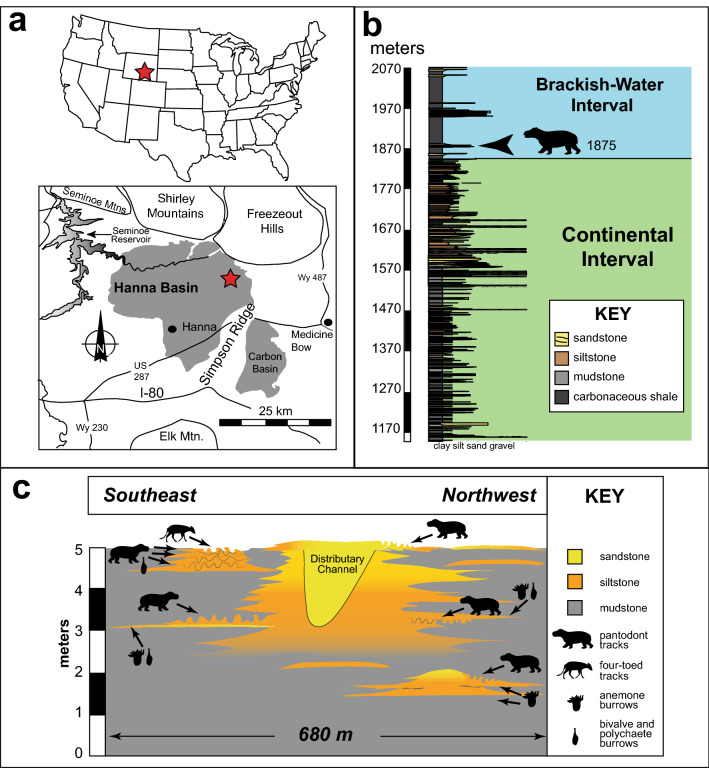
(**a**) Location and stratigraphic setting of the tracksite; (**b**) Measured section is stratigraphic elevation above local base of the Hanna Formation. Entire succession falls within P5 palynostratigraphic zone, with tracksite in the uppermost portion of the zone; (**c**) Stratigraphic and aerial distribution of mammal prints and other significant trace fossils within the main section of the tracksite. Drawings and maps made by A F-J W in Adobe Illustrator 2021 (version 25.2.1, https://www.adobe.com/products/illustrator.html). Photographs by A F-J W and compiled in Microsoft PowerPoint (version 16.0.13801.20266, https://www.microsoft.com/en-us/microsoft-365/powerpoint).

The Paleocene-Eocene (63–53 Ma) Hanna Formation in the Hanna Basin (Wyoming, U.S.A.) contains a mosaic of fluvial, lacustrine, and brackish-water paleoenvironments^5,6^. The track-bearing interval occurs in the lower portion of a newly recognized, 216 m thick, brackish-water member of the formation (Fig. 1). Fossil plants and pollen collected from surrounding deposits indicate a late Paleocene age (upper P5-P6 palynostratigraphic zone: 58 Ma) for the tracksite^6^. The bulk of the formation below the brackish-water unit consists of siltstone, fine-grained sandstone, and pebble to cobble conglomerate weathering into badlands topography. Crayfish burrows preserved within siltstones demonstrate seasonally low paleo-water tables and the development of well-drained soils^7^. Transition to the unnamed, brackish-water member is marked by a lithological shift to carbonaceous shales, coals, and prominent, rusty-orange sandstone and siltstone ridges. This unit is further differentiated by the presence of definitively marine ichnofossils, including *Arenicolites, Bergaueria, Cylindrichnus, Gyrochorte, Ophiomorpha, Palaeophycus, Rhizocorallium, Siphonichnus, Skolithos, and Thalassinoides*, preserved in silty and very fine-grained sandy delta front, tidal flats, and lagoonal deposits.

Hundreds of prints are preserved in at least five discrete horizons within two separate, silty to very fine-grained sandy delta lobes (Fig. 1c). The delta complex is 12 m thick, with individual lobes composed of 1–2 m thick packages of coarsening-upward clay, silt, and very fine-grained sand. A 1.5–2 m thick and 113 m wide distributary channel separates the northwestern portion of the trackway from the southeastern. Wavy and lenticular bedding dominate prodelta and delta front successions with current ripples, climbing ripples, and small-scale trough and planar cross-bedding more common towards the proximal delta front. In addition to the mammal footprints, heavily-burrowed, contorted siltstone beds are marked by an abundance of polychaete and bivalve traces, supporting their interpretation as tidal flat environments. The presence of *Bergaueria* (sea anemone burrows)*, Rhizocorallium* (polychaete burrows)*, Gyrochorte* (polychaete trails)*, Siphonichnus* (marine bivalve burrows), and other marine-derived ichnofossil suites throughout the silty beds is definitive evidence of marine or brackish-water influence during deposition of these strata^8–15^.

## Description of tracks and trackways

**Figure 2:**
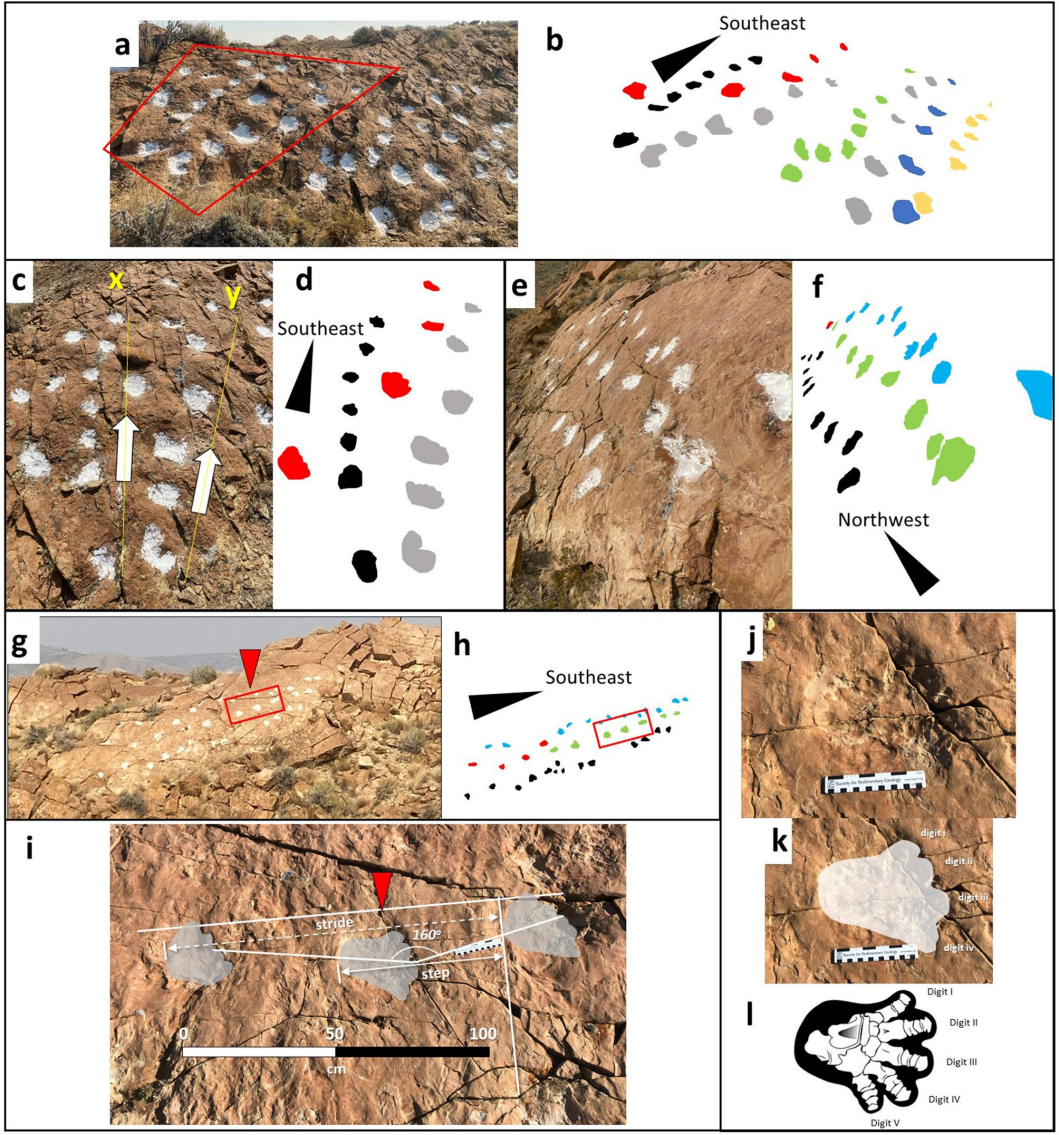
Representative surface expression of trackways. (**a**) Photograph of heavily trampled surface of the uppermost tracksite at the southern end of the outcrop; (**b**) Interpretation of individual trackways; (**c**) Photograph of section of tracksite outlined by polygon in **a**. Tracks to the left of line X are from a smaller individual. Tracks to left of line Y are from a larger individual; (**d**) Interpretation of individual tracks color coded as in **b**; (**e**) Photograph of northern expression of the uppermost trackway; (**f**) Interpretation of individual tracks depicted in **e**; (**g**) Photograph of surface of outcrop with four trackways highlighted by baking flour; (**h**) Interpretation of trackways in **h**; (**i**) Photograph of representative series of three consecutive tracks in a narrow-gauge trackway from area highlighted by rectangle in **g** and **h** showing step, stride, and pace angulation of 160°; (**j**) Photograph of middle track noted by arrow in **g**–**i**; (**k**) Interpretive outline of **J** with digits labelled; (**l**) Illustration of skeletal elements of *Coryphodon radians* pes (modified from original^18^) for comparison with **j** and **k**. Drawings made by A F-J W in Adobe Illustrator 2021 (version 25.2.1, https://www.adobe.com/products/illustrator.html). Photographs by A F-J W and compiled in Microsoft PowerPoint (version 16.0.13801.20266, https://www.microsoft.com/en-us/microsoft-365/powerpoint).

**Figure 3:**
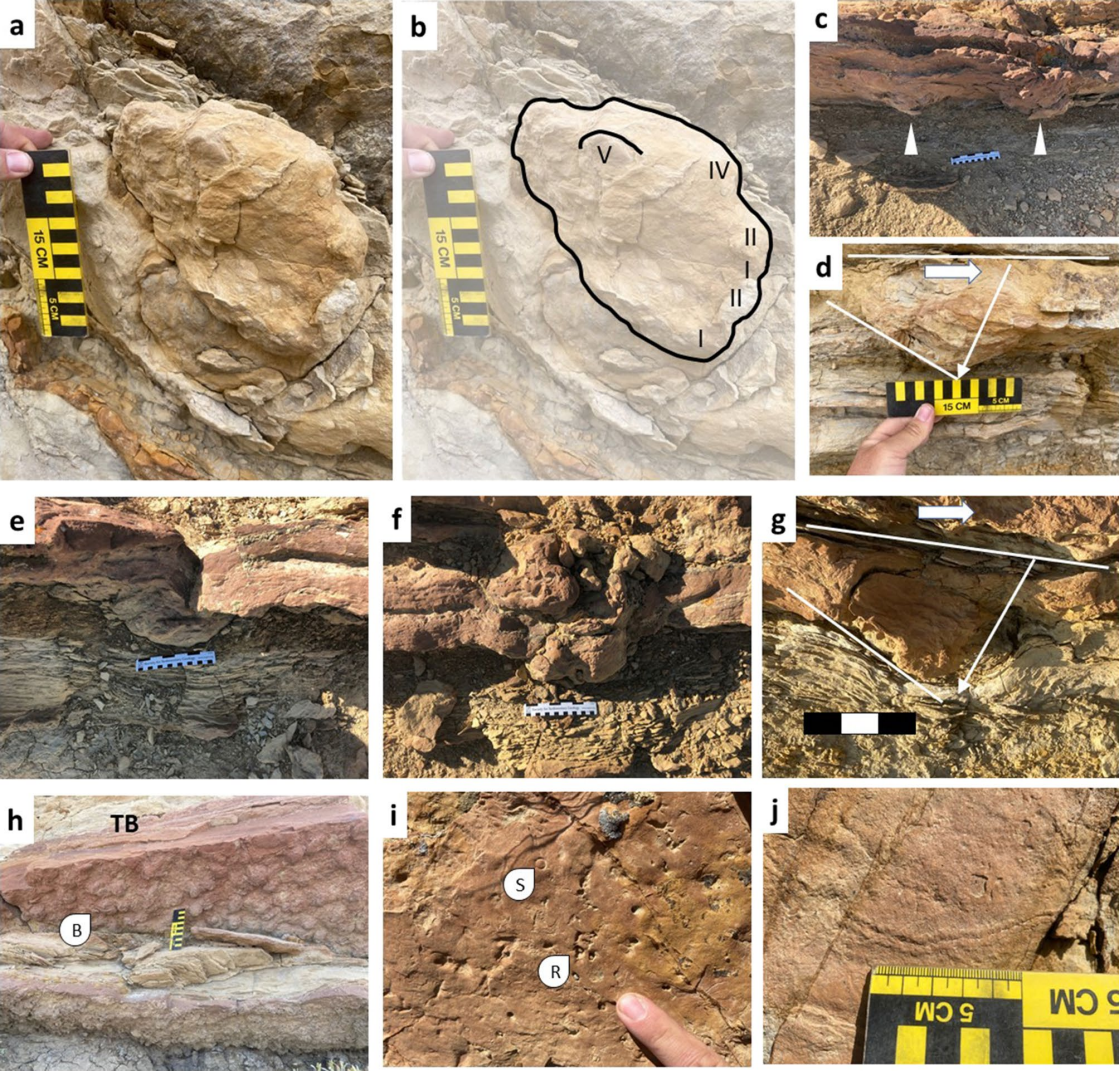
Photographs of representative examples of penetrative prints and associated brackish-water ichnofossils. Scale bars are 15 cm. (**a**) Natural cast of blunt-toed print left by animal walking left to right (southeast to northwest); (**b**) Interpretive outline of A; (**c**) Consecutive footsteps of an animal walking left to right (southeast to northwest),arrows indicate penetration of footprints; (**d**) Print made by animal walking right to left (southeast to northwest), small arrow indicates downward rotation of foot during step-off; (**e**) Underprint in silty claystone 20 cm below sandstone bed; (**f**) Penetrative print with underprint in silty claystone and deformation in track-bearing sand; (**g**) Natural sandstone cast and penetrative print exhibiting downward rotation of animal walking left to right (southeast to northwest); (**h**) Underside of a track-bearing silty sandstone bed (TB) with abundant *Bergaueria* (B) at the base, overlying a siltstone bed with additional *Bergaueria* at the base; (**i**) Upper surface of silty sandstone bed with abundant, small *Rhizocorallium* (R) and *Siphonichnus* (S) among other marine ichnofossils. Index finger of AF-JW for scale; (**j**) *Gyrochorte* in very fine-grained sandstone bed underlying track-bearing siltstone. Photographs by A F-J W and compiled in Microsoft PowerPoint (version 16.0.13801.20266, https://www.microsoft.com/en-us/microsoft-365/powerpoint).

**Figure 4:**
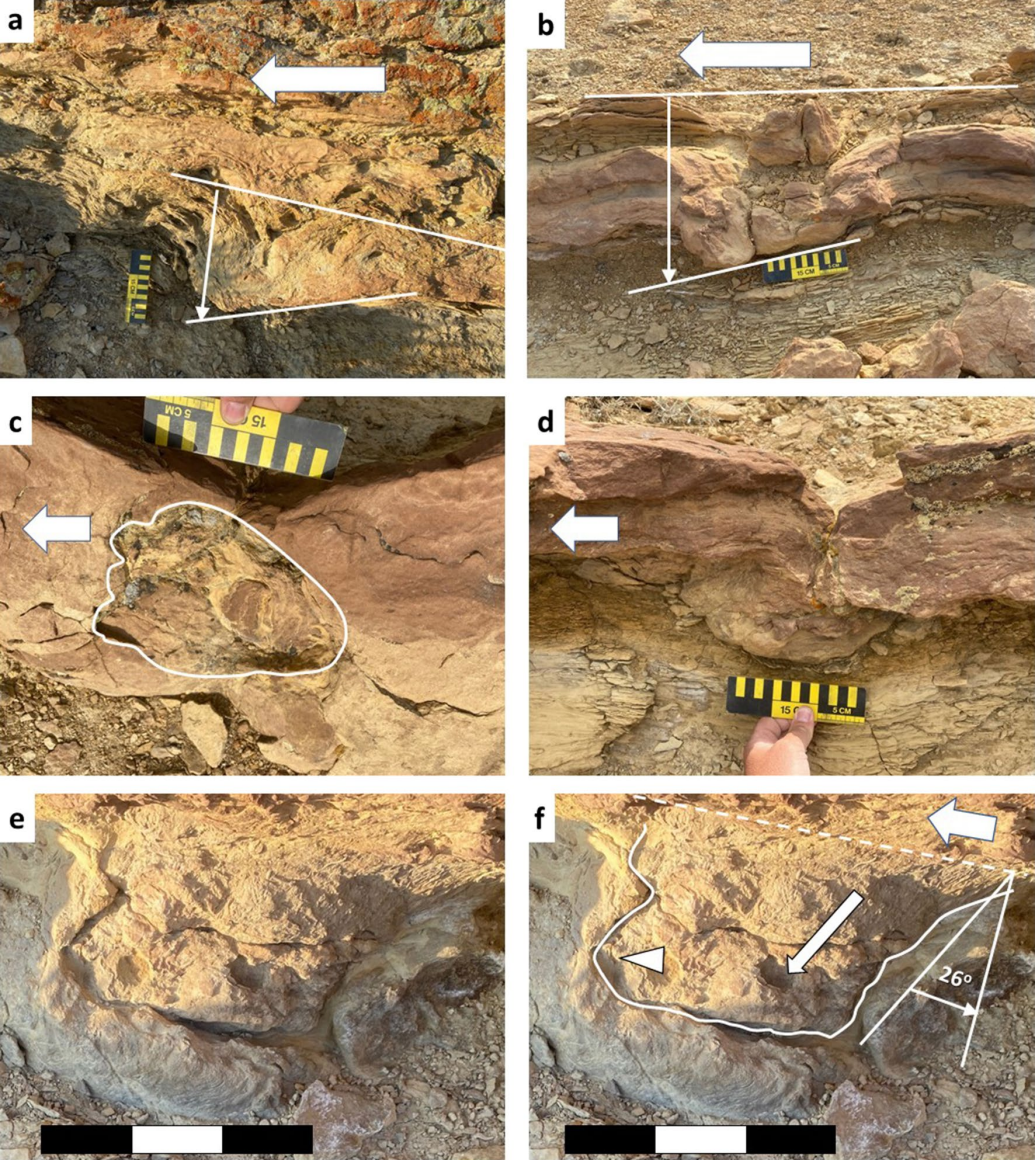
Photographic evidence of dynamic locomotion in tracks. **a**, **b**, **d**–**f** are in cross-section. **c** is surface view. Scale bars are 15 cm. Large arrows indicate direction of travel (southeast). (**a**) Downward rotation of anterior part of the pes and digits; (**b**) Downward rotation with disruption of underlying, heterolithic bedding; (**c**) Pes with narrow heel and poorly preserved digit impressions; (**d**) Cross sectional view of **c** showing clear deformation of underlying sediment and penetration into underlying, heterolithic bedding; (**e**) Example of deep pes track with natural cast exposed in cross-sectional view; (**f**) Interpretive outline of **e**. Small arrow indicates angle of impact during step-in (26° from vertical).Triangle indicates expansion of digits into substrate with moderate downward rotation. Drawings made by A F-J W in Adobe Illustrator 2021 (version 25.2.1, https://www.adobe.com/products/illustrator.html). Photographs by A F-J W and compiled in Microsoft PowerPoint (version 16.0.13801.20266, https://www.microsoft.com/en-us/microsoft-365/powerpoint).

**Figure 5:**
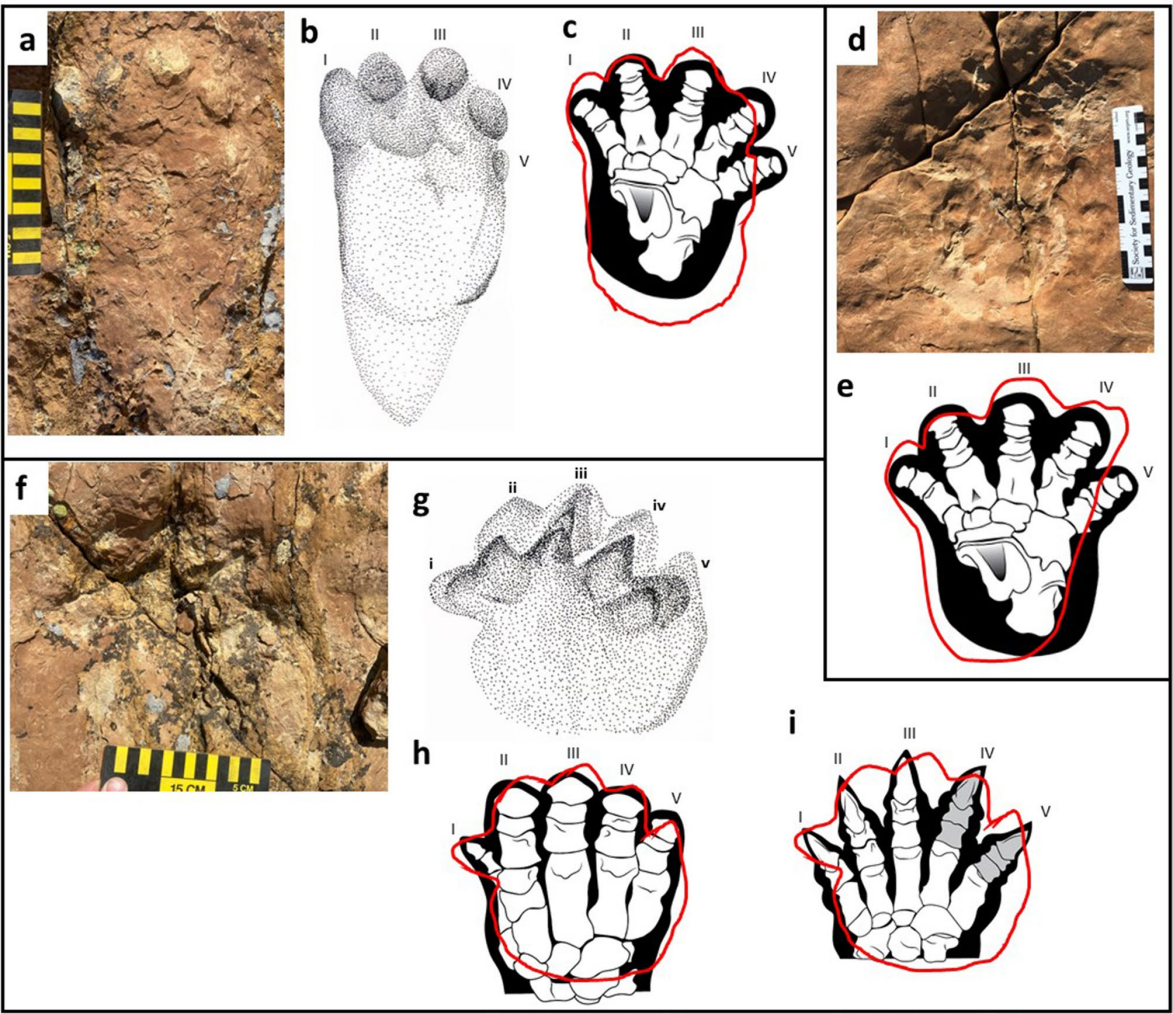
Representative examples of individual five-toed footprints with clear toe impressions. Scale bars are 15 cm. (**a**) Photograph of five-toed pes; (**b**) Interpretive sketch of **a** with digits numbered; (**c**) Illustration of skeletal elements of *Coryphodon radians* pes (modified from original^18^) with outline of **a** superimposed; (**d**) Photograph of five-toed pes demonstrating deep penetration; (**e**) Illustration of skeletal elements of *C.* *radians* pes (modified from original^18^) with outline of **d** superimposed; (**f**) Photograph of five-toed print identified as a manus or possible direct register pes imprint; (**g**) Interpretive sketch of **f** with digits numbered; (**h**) Illustration of skeletal elements of *C.* *radians* manus (drawn from mounted specimen) with outline of **f** superimposed; (**i**) Illustration of skeletal elements of *Titanoides primaevus* manus with missing bones in grey (modified from original^19^) with outline of **f** superimposed. Drawings made by A F-J W in Adobe Illustrator 2021 (version 25.2.1, https://www.adobe.com/products/illustrator.html). Photographs by A F-J W and compiled in Microsoft PowerPoint (version 16.0.13801.20266, https://www.microsoft.com/en-us/microsoft-365/powerpoint).

**Figure 6:**
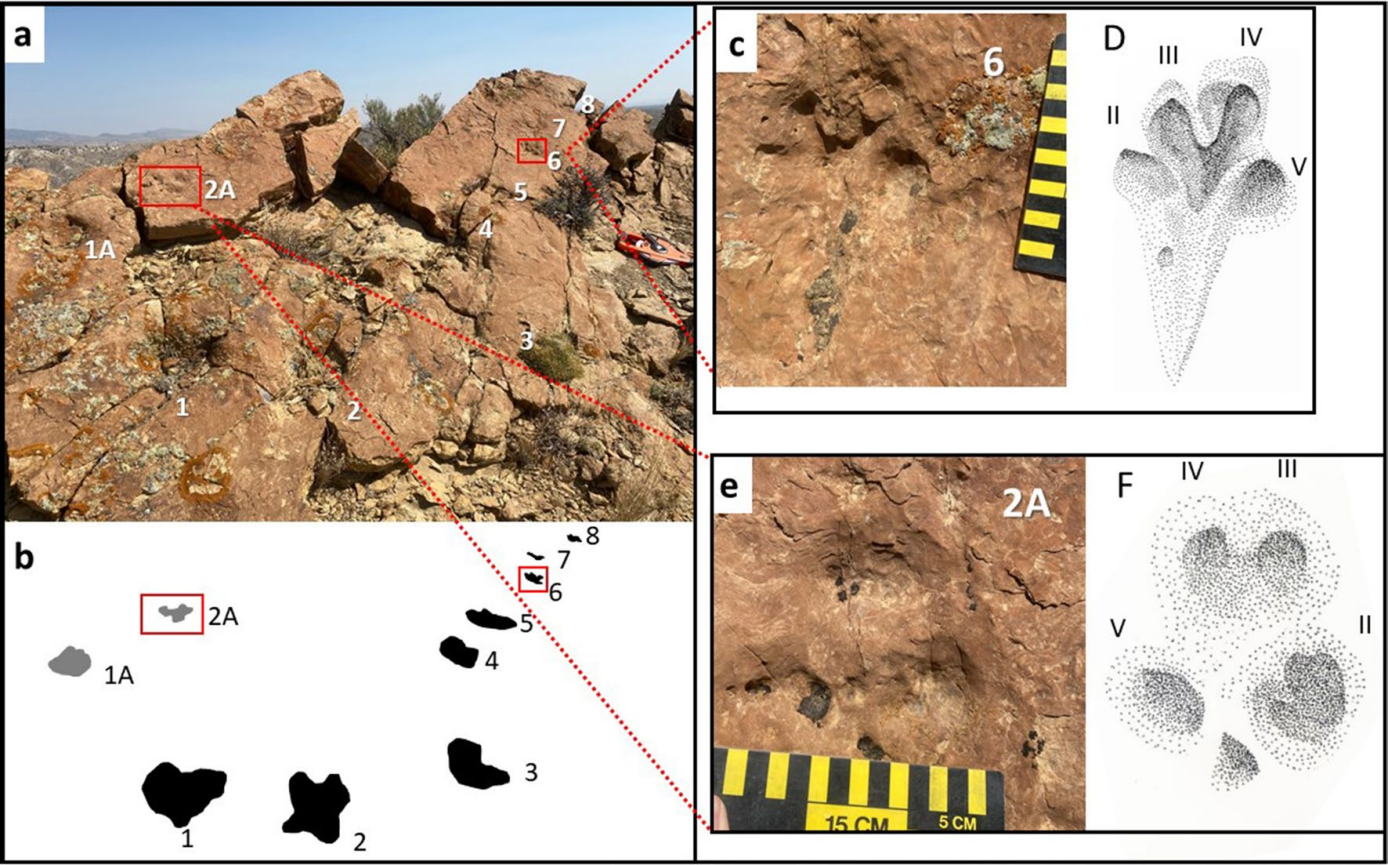
Representative examples of four-toed prints. Scale bars are 15 cm. (**a**) Photograph of trackways. Numbers correspond to individual tracks and red boxes indicate location of tracks figured in **c**–**f**; (**b**) Interpretive outline of individual tracks in **a**; (**c**) Photograph of four-toed print (6 from **a** and **b**) demonstrating slide-in; (**d**) Interpretive sketch of **c** with digits numbered; (**e**) Photograph of four-toed print (2A from **a** and **b**) with middle digits closely appressed, creating the appearance of three digits; (**f**) Interpretive sketch of **e** with digits numbered. Drawings made by A F-J W in Adobe Illustrator 2021 (version 25.2.1, https://www.adobe.com/products/illustrator.html). Photographs by A F-J W and compiled in Microsoft PowerPoint (version 16.0.13801.20266, https://www.microsoft.com/en-us/microsoft-365/powerpoint).

**Figure 7:**
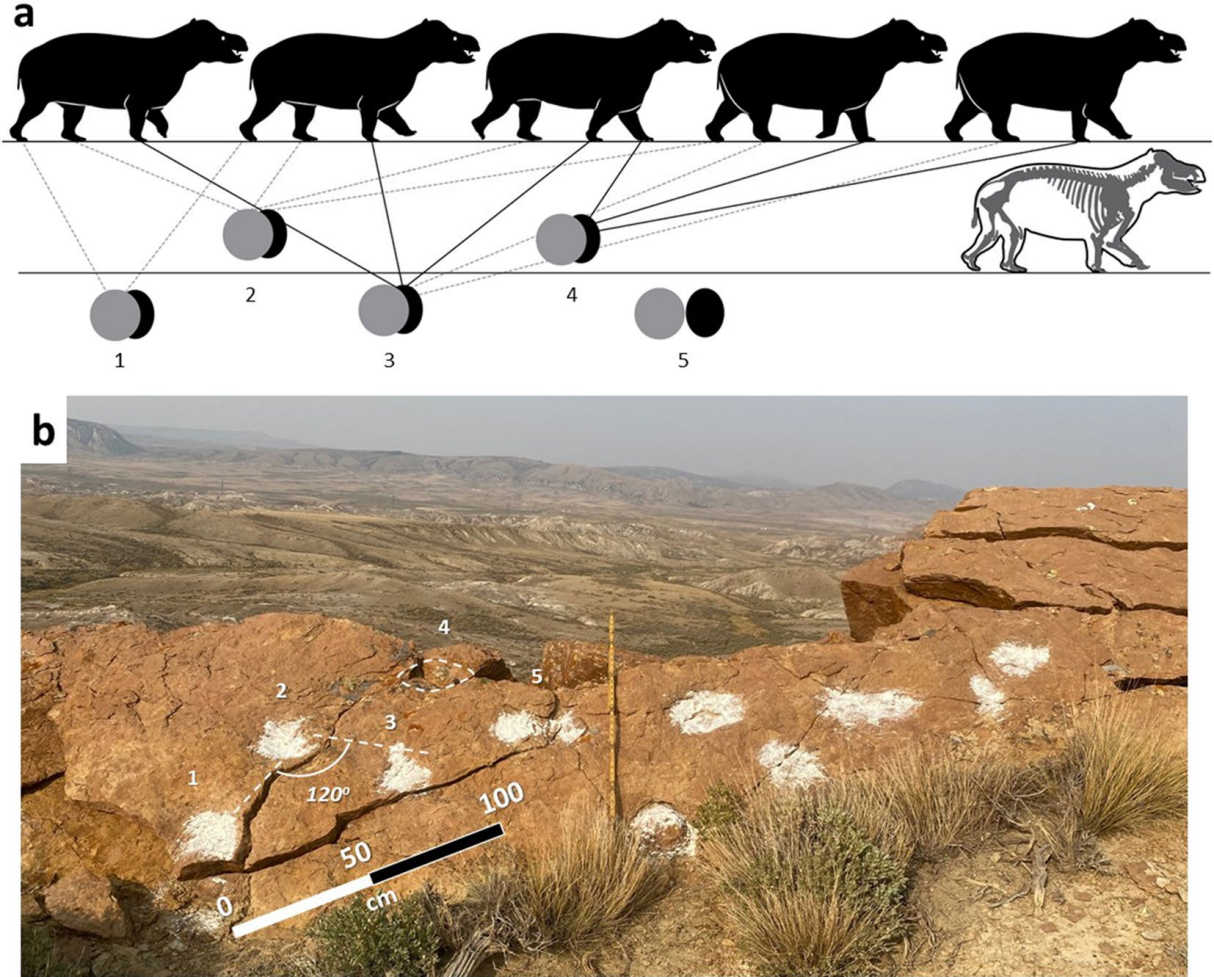
Example of wide gauge trackway and candidate track maker. (**a**) Walk cycle of *Coryphodon* inferred from trackway showing slow, direct register to understep walk. Biomechanical reconstruction was constrained by skeletal elements preserved for *C. radians* (modified from original^18^) as depicted at lower left; (**b**) Photograph of trackway from which **a** is modeled (view to the northeast). Scale bar is 1 m. Drawings made by A F-J W in Adobe Illustrator 2021 (version 25.2.1, https://www.adobe.com/products/illustrator.html). Photographs by A F-J W and compiled in Microsoft PowerPoint (version 16.0.13801.20266, https://www.microsoft.com/en-us/microsoft-365/powerpoint).

Along the 1032 m transect of track-bearing strata, four print morphotypes can be recognized: (1) round, amorphous depressions on bedding surfaces, which have visible underprints where exposed in cross-section (Figs. 2, 3, 4, 5, 6); (2) moderately- to well-preserved, five-toed prints preserved on bedding surfaces (Figs. 2, 5, and 7); (3) well-preserved, four-toed prints preserved on bedding surfaces (Fig. 6); and (4) natural casts penetrating underlying, heterolithic strata and exposed in cross-sectional view with limited or no surface exposure (Figs. 3 and 4). These trackways are distinguishable from erosional features, stingray feeding pits, and invertebrate traces based on the following characteristics: (1) where visible, sedimentary layers within and below the individual tracks are clearly and consistently compacted and displaced downward to varying degrees, as has been documented in vertebrate walking traces in which the dynamic load of the maker’s foot shifts throughout the step cycle^3,16^ (Figs. 3 and 4); (2) clear, individual toe impressions are discernable in seven of the surface tracks and one of the natural casts (Figs. 2j–l, 3a,b, 5, and 6), while other tracks demonstrate less-defined digit impressions along their leading edge; and (3) the ichnofossils exhibit a regular and uniform pattern of size and spacing, typical of vertebrate walking gaits^17^ (Figs. 2 and 7).

Siltstone beds contain prints preserved with clearer outlines and digit impressions than those in fine-grained sandstone beds. The lower surface area to volume ratio of individual sand grains compared to silt results in lower grain-to-grain adhesion and a decreased ability for the substrate to retain an imprint, causing prints to be poorly preserved in sandstone compared to those in siltstone beds.

Individual five-toed imprints are mostly poorly defined, but measure on average between 15–20 cm long and 15–22 cm wide with specimens ranging up to 24.5 cm long and 25 cm wide, approximating the upper size range of the North American Brown Bear (*Ursus arctos*)^17^ (Figs. 2j–l and 5). Four larger toes are directed forward, while a smaller, fifth toe is angled nearly 90° to the others or fails to register (Fig. 5a, c–e). Two of the four clearest bedding plane prints are broader than they are long (21.5 × 15 cm and 19 × 14 cm), one is equally broad as it is long (18 × 18 cm), and a fourth appears longer than it is broad (37 × 15 cm), but exact measurements of the latter are hampered by the incomplete register of the foot along its posterior margin and possible slide-in mark (Fig. 5a,b). A particularly deep track preserves possible evidence of a direct register gait in which the apparently narrow-clawed manus impression is partially or completely obliterated by the pes (Fig. 5f,g). Alternatively, the imprint may be an artifact of the deep penetration and withdrawal of a blunt-toed foot triggering inward collapse of more saturated, deeper sediment, giving the impression of narrower digits towards the bottom of the print.

Clearly distinguishable from the larger, five-toed tracks are prints bearing four distinct, equally-sized digits (Fig. 6). One 11 cm-wide × 11.5 cm-long print with four toe imprints exhibits a drag mark representing forward and downward movement of the foot (Fig. 6c,d). A possible partial imprint of another, forward-facing digit is visible but is obscured by the third digit from the left, a pattern consistent with a direct register gait. The print is situated within a trackway consisting of eight amorphous prints with a bearing of 94°. A roughly parallel trackway (bearing 97°) of four prints 58 cm to the north of these tracks also includes a four-toed imprint (Fig. 6e,f). The two middle digits in this 13 cm-wide × 12 cm-long track are tightly appressed and initially appear to be a single digit. Both four-toed prints were likely made by the same mammalian taxon, and differences in the spread between the median digits of the prints in each trackway resulted from varying digit placement, probably reflecting differences in substrate moisture. It is also possible that the two prints represent a manus and pes and, therefore, display subtle differences in digit position.

Where exposed in cross-section, the larger, five-toed prints display key characteristics of tracks made by heavy vertebrates in water-saturated, weakly-consolidated sediment^16,20,21^(Figs. 2, 3, 4, 5, 6). Although poor preservation precludes detailed analysis of print morphology in the cross-sectional examples, evidence for the direction of travel is afforded by the downward rotation of the anterior portion of the pes or manus as the digits press deeper into the substrate during forward propulsion^3^. Additional locomotory information can be gleaned from the 26° angle of impact and preservation of digit expansion evident in some of the cross-sectional tracks (Fig. 4e,f). Distinct digit imprints are rare in deeply penetrating footprints and natural casts; however, five blunt digit marks can be recognized in a single 17.25 cm-long natural cast (Fig. 3a,b) and less distinct impressions are distinguishable in several other penetrative tracks (Fig. 4c).

The stratigraphically-lowest trackway is located to the northwest of the distributary channel (Fig. 1c). A broadly lenticular, silty sandstone bed hosts thousands of *Bergaueria* at its base (Fig. 4h). Polychaete and bivalve burrows (*Skolithos* and *Siphonichnus*, respectively) are distributed across the surface of this bed, indicating continuation of marine conditions after deposition of the sediment (Fig. 4i). Mammalian prints originate in a siltstone horizon 25–30 cm above the base of the bed and penetrate as deep as 39 cm into the underlying silty claystone (Fig. 4f). Tracks are exposed in plan-view as well as in cross-section with several preserved in both views, confirming that surface-only tracks are not simply anomalous depressions (Fig. 5c,d).

Southeast of the distributary channel, a continuous, 356 m-long exposure of several trackways exhibits planform and cross-sectional imprints that penetrate and deform underlying strata (Fig. 2a–d). Four separate horizons are identifiable, each representing flood-delivery of siltstone and very fine-grained sand onto the brackish-water delta front, mouth bars, and tidal flats. The uppermost horizon is traceable for 230 m and contains hundreds of individual prints arranged into more than 20 distinct trackways, although portions are obscured by vegetation cover or damaged by weathering. The majority of prints with discernable digit impressions are located in this horizon, their enhanced preservation attributable to the rheology of the very fine siltstone in which these tracks were imprinted. Evidence of social behavior might exist in some of the clearer trackways. For instance, a pair of tracks consisting of a large (average = 17.5 cm wide × 14.8 cm long) and a 70% smaller (average = 12 cm wide × 11 cm long) set of prints, walking in tandem to the southeast (105° bearing) might represent an adult and juvenile or a sexually-dimorphic pair traversing together for approximately 3 m (Fig. 2c,d).

A well-exposed, 7 m-long, planform trackway composed of amorphous and five-toed morphotypes is located proximal to the northwestern side of the distributary channel and includes parallel tracks of at least four individuals preserved in very fine-grained sandstone (Fig. 2e–h). This trackway appears to be the highest stratigraphically, but the distributary channel forms a stratigraphic barrier that impedes its direct correlation to any of the trackways to the southeast (Fig. 1). All the trackways in this exposure are narrow-gauge with a pace angulation of 160° (Fig. 2i) in contrast to those on an exposure of the same surface, 40 m to the northwest, where pace angulation is 120° (Fig. 7). In comparison, trackways of *Thulitheripus svalbardii* exhibit pace angulations of 113°, 118°, and 125°^3^. Narrowing of trackway width concurrent with lengthening of individual steps occurs when quadrupeds increase walking speed^17^. Accordingly, the wider gauge and shorter step length of the tracks to the north and the narrow-gauged trackways with longer steps in the south suggest animals may have been ambulating at different speeds across the region. Evidence for a direct register walking pattern is found in many of the trackways where clear manus-pes couplets are indiscernible. Common among quadrupedal mammals, this gait results from placement of the pes directly upon the manus imprint^17^ (Fig. 7).

## Discussion

### Identity of track makers

**Figure 8:**
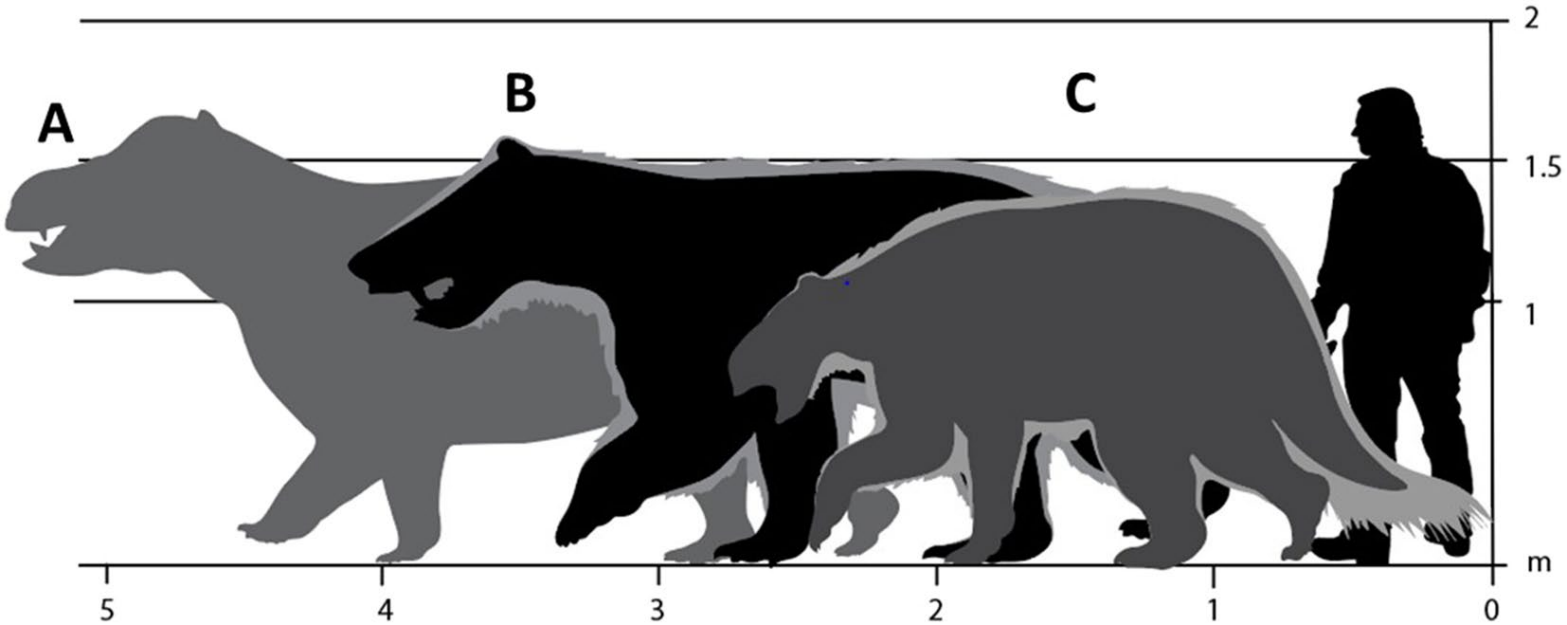
Reconstructed track maker candidates for size comparison. (**A**) Paleocene *Coryphodon proterus* (Yale Peabody Museum specimen VPOU.016130, with a 62 cm long skull), based on measurements of Eocene *C. radians*^18^; (**B**) *Titanoides primaevus* based on skeletal reconstruction^19^ and scaled to holotype specimen (Yale Peabody Museum VPPU.016490); (**C**) *Barylambda faberi* based on mounted specimen displayed at the Field Museum of Natural History. Drawings made by A F-J W in Adobe Illustrator 2021 (version 25.2.1, https://www.adobe.com/products/illustrator.html).

The late Paleocene age of the Hanna Formation trackways limits the known mammalian taxa sizeable enough to produce the five-digited prints to the Pantodonta^3,18^. One or more species, such as *Titanoides primaevus, Barylambda faberi,* or *Coryphodon proterus*, were likely responsible for the tracks deposited in this Paleocene lagoon (Fig. 8). *Titanoides* was identified as the originator of Late Paleocene *Thulitheripus svalbardii* from Norway^3^. The five clear digit impressions of *T. svalbardii,* with those on the manus appearing to be claw-like and strongly curved, bear some resemblance to the deeper print from the possible direct register track described above (Fig. 5f). However, we cannot definitively attribute any of the tracks to *Thulitheripus* nor to its associated *Titanoides* maker since the apparently narrow-clawed manus imprint from the Hanna Formation may be an artifact of substrate collapse in the deeper portion of the track.

The brackish-water setting of the trackways and their recurrence of deposition suggests that their five-toed mammalian originators routinely exploited aquatic habitats. The morphology and stable isotope chemistry of *Coryphodon* indicate that this pantodont exhibited semiaquatic habits analogous to those of extant common hippopotamus (*Hippopotamus amphibius*)^21^. At an estimated weight of 700 kg, the only known Paleocene species of *Coryphodon*, *C. proterus*, was larger than later species and has only been documented from western North America^22^. The combination of mass and hypothesized natural history of these pantodonts renders them the most plausible candidates for our track makers. Since the Hanna Formation trackways are approximately 1.0–1.5 m.y. older than the earliest skeletal record of *Coryphodon*, confirmed attribution of these tracks to this genus would extend its origin farther into the Paleocene than indicated by the body fossil record alone.

The four-toed prints do not correspond to the skeletal elements of any mammals described from the late Paleocene but bear remarkable resemblance to tracks made by medium- to large-bodied artiodactyls and forefoot imprints of modern tapirs (Perissodactyla). Trace fossil evidence for the origination of animal taxa prior to their sampling in the body fossil record has been well documented for various invertebrate lineages, though not without controversy^23–25^. The earliest osteological record of tapiroids is from early Eocene strata of Ellesmere Island, Canada, fueling the hypothesis that the lineage had an earlier origin within North America^26^. Although the phylogeny of Paleocene mammals remains poorly constrained^27^, recent time-calibrated molecular investigations have proposed a late Cretaceous or early Paleocene (74.4–64.1 Ma, mean 67.7 Ma) origin for Cetartiodactyla and its sister taxon the Perisodactyla^28^. Consequently, attribution of the Hanna Formation four-toed tracks to either tapiroid or artiodactyl tracemakers is consistent with molecular phylogenetic studies and substantiates these hypotheses with tangible, physical evidence. Therefore, we contend that these four-toed Paleocene prints are attributable to as-of-yet undiscovered North American tapiroids or large-bodied artiodactyls.

Paleoecological Implications.

The restricted marine setting of the Hanna Formation tracks has intriguing paleoenvironmental and behavioral implications. The trackways demonstrate shallow, tidally influenced, brackish-water habitat use by at least two mammalian taxa during the late Paleocene (58 Ma), an interaction with marine environments previously unknown prior to the Eocene (48.6–37.8 Ma) on the basis of body fossils. Although the presence of sea anemone traces is typically associated with offshore or nearshore marine settings^29^, anemones can tolerate a wide range of physicochemical extremes, including subaerial exposure during low tide^30^. Therefore, the sea anemone traces abundantly preserved in beds bearing mammalian prints are consistent with shallow subtidal to intertidal settings (Fig. 3h).

The use of brackish-water habitats by modern large-bodied, terrestrial to semiaquatic mammals is relatively common. Mammals weighing ≥ 28 kg exploit estuarine and marine habitats temporarily to permanently, exhibiting a variety of non-exclusive behavioral strategies. Saltwater environments serve as dispersal routes between offshore islands or between mainland and island habitats for Asian elephants (*Elephas maximus*), Baird’s tapirs (*Tapirus bairdii*), caribou (*Rangifer tarandus*), chamois (*Rupicapra* spp.), Sumatran serow (*Capricornis sumatraensis*), and various species of deer^31–37^. The use of brackish-water environments may also reflect seasonally-expressed migratory responses to climatic stimuli, as demonstrated by the movement of polar bears (*Ursus maritimus*) with sea ice patterns and the rainy season dispersal of riverine hippopotamus to coastal regions^38,39^. Regular and year-round residence in estuarine and marine locales are generally associated with unique foraging opportunities provided in the form of saltwater-tolerant vegetation, invertebrates, vertebrates, and scavenge-able carcasses transported downstream by flooding events or landward by tidal action^40,41^. Aquatic habitats are also important for mammals seeking refuge from biting insects and/or potential predators^35,42^.

In subtropical to tropical regions, similar to the climatic and environmental conditions in which the Paleocene tracks were deposited, extant megafauna use brackish water environments for thermoregulation^35,42,43^. Hippopotamus submerge in South Africa’s St. Lucia Estuary to minimize sun exposure, regulate body temperatures, and reduce energy expenditure^43^. Estuarine areas shallow enough to stand in, but deep enough to remain partially to fully submerged (i.e., water depths of 1.0–1.49 m for animals with a shoulder height of 1.5 m), are preferred. Locations adjacent to river mouths support greater hippopotamus densities due to the opportunity for more efficient thermoregulation provided by bathymetric changes associated with sandy mouth bars^43^. Notably, the trackways we describe occur in sediments analogous to these microenvironments favored by thermoregulating hippopotamus. Specifically, the greatest density of tracks is situated proximal to the Paleocene distributary mouth (Fig. 1). Abundance of tracks decreases 412 m to the southeast of the channel, where exposure becomes limited, and disappears completely 300 m to the northwest of the channel despite good exposure.

Modern mammals are also drawn to brackish and marine environments to satisfy physiological requirements related to sodium deficiencies^35,42^. Forest elephants (*Loxodonta cyclotis*) in Gabon are attracted to salt-laden coastal vegetation, while Asian elephants in Malaysian rainforests frequent the coast to drink saline and hypersaline water^42,44^. Similar to their modern large, terrestrial mammal analogs, the Paleocene trackmakers were likely prone to mineral deficiencies due to excessive mineral-leaching from the adjacent tropical forest soil and the resulting decreased availability of sodium in the tropical and subtropical vegetation, driving them to take advantage of coastal mineral resources.

## Conclusions

We report the world’s largest assemblage of Paleocene mammal tracks preserved in Wyoming’s late Paleocene Hanna Formation. Not only are these trackways the first such reported for the United States, but they are only the fourth mammalian trackways described in the world. Hundreds of individual prints, representing at least two large mammalian taxa, are exposed in varying preservation in a minimum of five discrete horizons, which can be traced laterally for up to 1032 m.

Pantodonts are likely responsible for the larger (15–24.5 cm long × 15–25 cm wide), five-toed prints, with paleoecological data supporting the semi-aquatic *Coryphodon* as a probable candidate. Clear four-toed prints (11.5–12 cm long × 11–13 cm-wide) are also evidenced within the trackways, possibly left by as-of-yet undescribed artiodactyls or tapiroids. Molecular phylogenetic hypotheses for a late Cretaceous to early Paleocene origin of these taxa is thus significantly augmented by these ichnological data, despite the absence of osteological remains.

The trackways were deposited in a restricted marine embayment or lagoon, on silty and sandy tidal flats near an active fluvial distributary channel. This interpretation is supported by the presence of mammal tracks in beds hosting low-diversity, marine ichnofauna. Fossilized skeletal remains previously established the Eocene as the earliest marine habitat use by mammalian taxa, whereas the Hanna Formation trackways extend the origin for mammals’ expansion into brackish-water environments to at least 58 Ma. Late Paleocene mammalian megafauna were likely drawn to these ecosystems to fulfill similar needs as their modern analogues: migration, thermoregulation, protection from predators and biting insects, and access to sodium and other minerals, which would have been limiting in the North American Western Interior’s tropical forests. As such, the Hanna Formation trackways offer compelling insight into early Cenozoic mammalian evolution and paleoecology, providing us with a glimpse into the lives of megafauna mucking through the brackish-water tidal flats of the late Paleocene and preserving behavior that couldn’t be predicted from analysis of body fossils alone.

## Methods

Ichnological, stratigraphic, and sedimentological reconnaissance was undertaken for late Paleocene strata within the Hanna Formation, north of Medicine Bow Wyoming, USA from 2016–2020. Previously unidentified brackish-water strata and tracksites were subsequently identified, examined, measured, and photographed in the field. All-purpose baking flour was used to highlight the prints for photographic purposes, such that marking would not inflict any significant adverse impact on the natural environment. Sedimentary sections were logged through the stratigraphic interval comprising the deltaic complex in which the trackways are preserved. Grain size, sedimentary structures, bedding contact, lateral continuity, and associated trace fossils were recorded in detail. Individual beds were walked out to establish correlations and relationships to surrounding horizons. A variety of modern and historic footprints and tracksites were examined to critically evaluate the morphology, taxonomic affinity, and locomotory behavior of the trace-making taxa. Avian, reptilian, amphibian, and invertebrate animals were excluded as potential print-making candidates based on the morphology of the prints and the gait pattern preserved in many of the tracks. Black and brown bear (*Ursus arctos* and *U. americanus*, respectively) footprints were photographed and cast in dental plaster in Alaska and Colorado, USA, and Alberta, Canada for comparison with the Paleocene tracks.

## References

[1] McCrea RT, Pemberton SG, Currie PJ (2004). New ichnotaxa of mammal and reptile tracks from the Upper Paleocene of Alberta. Ichnos.

[2] Henderson DM (2015). A wide-gauge, large-mammal trackway from the upper Paleocene of Alberta Canada. Can. J. Earth Sci..

[3] Lüthje CJ, Milàn J, Hurum JH (2010). Paleocene tracks of the mammal pantodont genus *Titanoides* in coal-bearing strata, Svalbard, Arctic Norway. J. Vertebr. Paleontol..

[4] Davydenko S, Laime MJ, Gol'din P (2019). The earliest record of a marine mammal (Cetacea: Basilosauridae) from the Eocene of Amazonia. J. Vertebr. Paleontol..

[5] Hansen, D.E. Laramide tectonics and deposition of the Ferris and Hanna Formations, south-central Wyoming in *Paleotectonics and sedimentation in the Rocky Mountain Region, United States: American Association of Petroleum Geologists Memoir 41* (ed. Peterson, J.A.) 481–495 (AAPG, 1986).

[6] Dechesne M, Currano ED, Dunn RE, Higgins P, Hartman JH, Chamberlain KR, Holm-Denoma CS (2020). A new stratigraphic framework and constraints for the position of the Paleocene-Eocene boundary in the rapidly subsiding Hanna Basin, Wyoming. Geosphere.

[7] Hasiotis ST, Honey JG (2000). Paleohydrologic and stratigraphic significance of crayfish burrows in continental deposits: examples from several Paleocene Laramide basins in the Rocky Mountains. J. Sediment. Res..

[8] Gingras MK, Pemberton SG, Saunders TDA, Clifton HE (1999). The ichnology of modern and Pleistocene brackish-water deposits at Willapa Bay, Washington: variability in estuarine settings. Palaios.

[9] Gingras MK, Hubbard SM, Pemberton SG, Saunders T (2000). The significance of Pleistocene *Psilonichnus* at Willapa Bay, Washington. Palaios.

[10] Gingras, M.K., MacEachern, J.A., Dashtgard, S.E., Zonneveld, J.-P., Schoengut, J., Ranger, M.J., & Pemberton, G. Estuaries. in *Trace fossils as indicators of sedimentary environments. Developments in sedimentology, Volume 64* (eds. Knaust, D. &Bromley, R.G.) 463–507 (Elsevier, 2012).

[11] Gingras MK, MacEachern JA, Dashtgard SE, Ranger MJ, Pemberton SG (2016). The significance of trace fossils in the McMurray Formation, Alberta, Canada. Bull. Can. Pet. Geol..

[12] MacEachern, J.A., Bann, K.L., Bhattacharya, J., & Howell, C.D. Ichnology of deltas: organism responses to the dynamic interplay of rivers, waves, storms, and tides. in *River deltas-concepts, models, and examples, Volume 51, SEPM Special Publication* (eds. Giosan, L., & Bhattacharya, J.P.) 49–85 (SEPM, 2005).

[13] Hauk TE, Dashtgard SE, Pemberton SG (2011). Brackish-water ichnological trends in a microtidal barrier island–embayment system, Kouchibouguac National Park, New Brunswick, Canada. Palaios.

[14] Pemberton, S.G., & Wightman, D.M. Ichnological characteristics of brackish water deposits. in *Applications of ichnology to petroleum exploration. Volume 17, SEPM Core Workshops,* (ed. Pemberton, S.G.) 141–167 (SEPM, 1992).

[15] Hubbard SM, Gingras MK, Pemberton SG (2004). Palaeoenvironmental implications of trace fossils in estuary deposits of the Cretaceous Bluesky Formation, Cadotte region, Alberta, Canada. Fossils Strata.

[16] Xing L, Li D, Lockley MG, Marty D, Zhang J, Persons WS, You H, Peng C, Kümmell SB (2015). Dinosaur natural track casts from the Lower Cretaceous Hekou Group in the Lanzhou-Minhe Basin, Gansu, Northwest China: Ichnology, track formation, and distribution. Cretac. Res..

[17] Elbroch M (2003). Mammal tracks and sign.

[18] Osborn HF (1898). Evolution of the Amblypoda. Part I. Taligrada and Pantodonta. Bull. Am. Mus. Nat. Hist. Bull..

[19] Simons EL (1960). The Paleocene Pantodonta. Trans. Am. Philos. Soc..

[20] Bennett MR, Morse SA, Falkingham PL (2014). Tracks made by swimming Hippopotami: an example from Koobi Fora (Turkana Basin, Kenya). Palaeogeogr. Palaeoclimatol. Palaeoecol..

[21] Clementz MT, Holroyd PA, Koch PL (2008). Identifying aquatic habits of herbivorous mammals through stable isotope analysis. Palaios.

[22] Uhen MD, Gingerich PD (1995). Evolution of *Coryphodon* (Mammalia, Pantodonta) in the late Paleocene and early Eocene of northwestern Wyoming. Contrib. Mus. Paleontol. Univ. Michigan.

[23] Hasiotis ST (2004). Reconnaissance of Upper Jurassic Morrison Formation ichnofossils, Rocky Mountain Region, USA: paleoenvironmental, stratigraphic, and paleoclimatic significance of terrestrial and freshwater ichnocoenoses. Sed. Geol..

[24] Bordy EM, Bumby AJ, Catuneanu O, Eriksson PG (2009). Possible trace fossils of putative termite origin in the Lower Jurassic (Karoo Supergroup) of South Africa and Lesotho. S. Afr. J. Sci..

[25] Bromley RG, Buatois LA, Genise JF, Labandeira CC, Mngano MG, Melchor RN, Schlirf M, Uchman A (2007). Comments on the paper “Reconnaissance of Upper Jurassic Morrison Formation ichnofossils, Rocky Mountain Region, USA: Paleoenvironmental, stratigraphic, and paleoclimatic significance of terrestrial and freshwater ichnocoenoses” by Stephen T. Hasiotis. Sed. Geol..

[26] Eberle JJ (2004). A new ‘tapir’ from Ellesmere Island, Arctic Canada-implications for northern high latitude palaeobiogeography and tapir palaeobiology. Palaeogeogr. Palaeoclimatol. Palaeoecol..

[27] Halliday TJD, Upchurch P, Goswami A (2017). Resolving the relationships of Paleocene placental mammals. Biol. Rev..

[28] Zurano JP, Magalhães FM, Asato AE, Silva G, Bidau CJ, Mesquita DO, Costa GC (2019). Cetrtiodactyla: updating a time-calibrated molecular phylogeny. Mol. Phylogenet. Evol..

[29] Knaust D (2017). Atlas of trace fossils in well core: appearance, taxonomy and interpretation.

[30] Bingham BL, Freytes I, Emery M, Dimond J, Muller-Parker G (2011). Aerial exposure and body temperature of the intertidal sea anemone *Anthopleura elegantissima*. Invertebr. Biol..

[31] Jayewardene, J. *The elephant in Sri Lanka*. Wildlife Heritage Trust of Sri Lanka, Sri Lanka (1994).

[32] Miller, F.L. Inter-island water crossings by Peary caribou, south-central Queen Elizabeth Islands. *Arctic*, 8–12 (1995).

[33] Harveson PM, Grant WE, Lopez RR, Silvy NJ, Frank PA (2006). The role of dispersal in Florida Key deer metapopulation dynamics. Ecol. Model..

[34] Quigley DTG, Moffatt S (2014). Sika-like deer Cervus nippon Temminck, 1838 observed swimming out to sea at Greystones, Co., Wicklow: increasing deer population pressure?. Bull. Ir. Biogeogr. Soc..

[35] Castelló JR (2016). Bovids of the world: Antelopes, gazelles, cattle, goas, sheep, and relatives.

[36] Naranjo, E.J. Tapirs of the Neotropics. in *Ecology and conservation of tropical ungulates in Latin America* (ed. Gallina-Tessaro, S.) 439–451(Springer, 2019).

[37] Kavčić K, Corlatti L, Rodriguez O, Kavčić B, Šprem N (2020). From the mountains to the sea! Unusual swimming behavior in chamois *Rupicapra* spp. Ethol. Ecol. Evol..

[38] Roth HH, Hoppe-Dominik B, Mühlenberg M, Steinhauer-Burkart B, Fischer F (2004). Distribution and status of the hippopotamids in the Ivory Coast. Afr. Zool..

[39] Pilfold NW, McCall A, Derocher AE, Lunn NJ, Richardson E (2017). Migratory response of polar bears to sea ice loss: to swim or not to swim. Ecography.

[40] Smith TS, Partridge ST (2004). Dynamics of intertidal foraging by coastal brown bears in southwestern Alaska. J. Wildl. Manag..

[41] Lewis TM, Lafferty DJ (2014). Brown bears and wolves scavenge humpback whale carcass in Alaska. Ursus.

[42] Morgan BJ, Lee PC (2007). Forest elephant group composition, frugivory and coastal use in the Réserve de Faune du Petit Loango, Gabon. Afr. J. Ecol..

[43] Prinsloo AS, Pillay D, O’Riain MJ (2020). Multiscale drivers of hippopotamus distribution in the St Lucia Estuary, South Africa. Afr. Zool..

[44] Boonratana, R. A statewide survey to estimate the distribution and density of the Sumatran rhinoceros, Asian elephant and banteng in Sabah, Malaysia. Wildlife Conservation Society, New York (1997).

